# An HTLV-1-Infected Humanized Mouse Model Expressing HLA-A*02:01 Demonstrates Effective CTL-Mediated Suppression of HTLV-1

**DOI:** 10.3390/v17091249

**Published:** 2025-09-16

**Authors:** Shinsuke Nakajima, Motohito Goto, Sung-Il Lee, Tokifumi Odaka, Masaki Hino, Kenta Tezuka, Norihiro Takenouchi, Takaharu Ueno, Fhahira Rizkhika Admadiani, Riichi Takahashi, Isao Hamaguchi, Takeshi Takahashi, Mamoru Ito, Kazu Okuma

**Affiliations:** 1 Department of Microbiology, Faculty of Medicine, Kansai Medical University, Hirakata 573-1010, Japan; 2 Central Institute for Experimental Medicine and Life Science (CIEM), Kawasaki 210-0821, Japantakeshi-takahashi@ciea.or.jp (T.T.);; 3 Department of Model Animal, Institute of Biomedical Science, Kansai Medical University, Hirakata 573-1010, Japan; 4 Center for Next Generation Biologics Research, National Institute of Infectious Diseases, Japan Institute for Health Security, Tokyo 208-0011, Japan; 5 Department of Clinical Laboratory, Subaru Health Insurance Society Ota Memorial Hospital, Gunma 373-8585, Japan

**Keywords:** HTLV-1, humanized mouse, Tax-specific CTLs, HLA

## Abstract

Human T-cell leukemia virus type 1 (HTLV-1) establishes lifelong infection and is associated with severe diseases such as adult T-cell leukemia/lymphoma (ATL) and HTLV-1-associated myelopathy/tropical spastic paraparesis (HAM/TSP). Cytotoxic T lymphocytes (CTLs), especially those specific for the viral protein Tax, play a pivotal role in controlling HTLV-1 infection. However, conventional humanized mouse models fail to fully reconstitute human immune responses, limiting their utility for evaluating CTL-mediated immunity. This study aimed to establish a physiologically relevant in vivo model to investigate human CTL responses against HTLV-1. To achieve this, we utilized NOG-HLA-A02 transgenic (Tg) mice expressing human HLA-A02:01 on thymic epithelial cells, enabling proper development of HLA-restricted human T cells. Compared to conventional humanized NOG mice, HTLV-1-infected humanized NOG-HLA-A02 Tg mice exhibited significantly reduced HTLV-1 proviral load (PVL), decreased expansion of infected CD4^+^ T cells, a trend toward increased frequencies of Tax-specific CD8^+^ T cells, and prolonged survival. These results demonstrate that the expression of HLA-A02:01 facilitates robust CTL-mediated immune control of HTLV-1. This model provides a powerful platform for dissecting HTLV-1 immunopathogenesis and evaluating CTL-targeted therapeutic strategies, including vaccines and immune checkpoint inhibitors.

## 1. Introduction

Human T-cell leukemia virus type 1 (HTLV-1) is a retrovirus that primarily infects CD4^+^ T cells. Once acquired, the virus evades elimination by the host immune system and establishes lifelong persistence [1]. This chronic infection is the foundation for the development of two major diseases: adult T-cell leukemia/lymphoma (ATL) [2] and HTLV-1-associated myelopathy/tropical spastic paraparesis (HAM/TSP) [3,4]. A high proviral load (PVL) is a known risk factor for the development of both ATL [5] and HAM/TSP [6], underscoring the critical role of effective immune control in preventing disease progression.

Among the immune components involved in the control of HTLV-1, cytotoxic T lymphocytes (CTLs) play a central role in the surveillance and elimination of infected cells, including malignant ATL cells. Infected individuals commonly exhibit detectable levels of CTLs specific for Tax, a viral transactivator protein that is essential for viral replication/transcription and serves as a major target of host cellular immunity [7,8]. In asymptomatic carriers (ACs), the frequency of Tax-specific CTLs in peripheral blood inversely correlates with the PVL, indicating their involvement in viral control [9]. In contrast, patients with ATL exhibit significantly reduced frequencies of Tax-specific CTLs compared to ACs [10]. Moreover, Tax-specific CTLs in ATL patients display impaired effector functions, including reduced degranulation capacity and diminished cytokine secretion, compared to those in ACs [10,11]. ATL cells evade Tax-specific CTL responses through mutations in the tax gene or transcriptional silencing caused by deletions or CpG hypermethylation in the 5′ long terminal repeat promoter region [12,13,14]. Moreover, ATL cells expressing Foxp3 exhibit regulatory T-cell-like suppressive activity and may contribute to immune suppression [15]. Collectively, these findings suggest that Tax-specific CTLs play a role in preventing the onset and limiting the progression of ATL.

To advance our understanding of CTL-mediated immune responses and their role in HTLV-1 pathogenesis, physiologically relevant in vivo models are essential. Although HTLV-1 can infect non-human primates and rats, it does not naturally infect mice. To overcome this limitation, humanized mouse models, severe immunodeficient mice engrafted with human hematopoietic stem cells (HSCs), have been developed to support HTLV-1 infection and are widely used to evaluate antiviral therapies [16]. However, these models have an incompletely reconstituted human immune system and exhibit limited adaptive immune responses, including impaired antibody production and reduced CTL activity. As a result, they do not faithfully reproduce the human immune response to HTLV-1, particularly the antigen-specific cytotoxicity mediated by CTLs.

One key limitation of conventional humanized NOD.Cg-*Prkdc* ^scid^*Il2rg*^tm1Sug^/Jic mice (hereafter referred to as NOG) is that human T cells are positively selected by mouse thymic epithelial cells, resulting in T-cell receptors (TCRs) restricted to mouse MHC molecules [17]. This mismatch hampers appropriate recognition of human antigen–MHC complexes. To address this issue, NOD.Cg-*Prkdc*^scid^ *Il2rg*^tm1Sug^ Tg (HLA-A*0201/H2-K^b^) ^A0201^/Jic mice (hereafter referred to as NOG-HLA-A02 Tg) expressing human HLA-A*02:01 on thymic epithelial cells have been developed [18]. While NOG-HLA-A02 Tg mice have been shown to support the generation of allogeneic CTLs, other humanized HLA-A2 transgenic mouse models, such as humanized NOD-scid IL2rγ^null^ Tg (HLA-A2/Huβ2M) mice [19] and NSG-HLA-A2/HHD mice [20], have been used to evaluate human CTL responses.

In this study, we employed humanized NOG-HLA-A02 Tg mice to investigate how antigen-specific cytolytic activity influences HTLV-1 infection. This improved model enables the assessment of Tax-specific CD8^+^ T-cell responses in the context of HTLV-1 infection and may serve as a valuable tool for developing effective immunotherapeutic strategies.

## 2. Materials and Methods

### 2.1. Cells

Human umbilical cord blood was obtained from the Japanese Red Cross Kinki Cord Blood Bank (Osaka, Japan), with informed consent obtained from all participants. The HTLV-1-infected T-cell line JEX28 was derived from Jurkat cells and established in our laboratory, using the same methodology as previously described for the JEX22 cell line [21,22]. Cells were cultured in RPMI-1640 medium supplemented with 10% fetal bovine serum (HyClone Laboratories, Logan, UT, USA), 100 U/mL penicillin (Meiji Seika Pharma, Tokyo, Japan), and 100 μg/mL streptomycin (Meiji Seika Pharma) at 37 °C with 5% CO_2_.

### 2.2. Animals

NOG and NOG-HLA-A02 Tg mice (formally NOD.Cg-Prkdcscid Il2rgtm1Sug Tg(HLAA*0201/H2-Kb)A0201/Jic) [18] were obtained from the Central Institute for Experimental Medicine and Life Science, Kawasaki, Japan. Only female mice were used in this study. All mice were maintained under specific pathogen-free conditions with controlled temperature (21–23 °C) and humidity (40–60%), a 12 h light/dark cycle, and free access to food and water. The potential influence of sex on the results is currently unknown.

### 2.3. Antibodies

Fluorescent-conjugated antibodies used for flow cytometric analyses included anti-human CD3 (clone SK7, BioLegend, San Diego, CA, USA, RRID: AB_10640737), anti-human CD4 (clone RPA-T4, BioLegend, RRID: AB_389311), anti-human CD7 (clone CD7-6B7, BioLegend, RRID: AB_2650637), anti-human CD8 (clone OKT-8, Thermo Fisher Scientific, Waltham, MA, USA, RRID: AB_10548030), Biotinylated anti-human CADM1 (clone 3E1, MBL, Nagoya, Japan, RRID: AB_592783), and T-Select HLA-A02:01 HTLV-1 Tax11-19 Tetramer-LLFGYPVYV-PE (MBL, RRID: not available). Streptavidin–Phycoerythrin (BioLegend, RRID: not available) was used to detect the biotinylated CADM1.

### 2.4. Purification of Hematopoietic Stem Cells

Mononuclear cells (MNCs) were isolated from umbilical cord blood by density gradient centrifugation using Lymphoprep (Serumwerk Bernburg, Bernburg, Germany), following the manufacturer’s instructions. CD133^+^ cells were subsequently purified from the MNCs using a CD133 MicroBead Kit (Miltenyi Biotec, Tokyo, Japan), as per the manufacturer’s protocol. HLA-A typing was performed using a WAKFlow HLA Typing Kit (Wakunaga, Hiroshima, Japan), according to the manufacturer’s instructions.

### 2.5. Generation of Humanized Mice

Seven-week-old NOG and NOG-HLA-A02 Tg mice were subjected to sublethal irradiation with 2 Gy from a 137Cs source (Gammacell 40 exactor, Nordion Inc., Ottawa, ON, Canada). Sublethal irradiation is commonly applied before human HSCs transplantation because it enhances engraftment efficiency by reducing host hematopoietic cells and creating space in the bone marrow niche, thereby reducing competition and allowing donor HSCs to engraft and expand [23]. Within 24 h after irradiation, the mice received an intra-bone marrow injection (IBMI) of 3 × 10^4^ human CD133^+^ cells carrying the HLA-A*02:01 allele. These mice are derived from the NOD/SCID background and carry a knockout of the extracellular domain of the common γ chain (*Il2rg*), which prevents the typical leakiness of mouse-derived CD4^+^ T cells observed in NOD/SCID mice, allowing engrafted human immune cells to dominate, consistent with previous reports [24].

### 2.6. HTLV-1 Infection

The HTLV-1-infected T-cell line JEX28 was irradiated with 10 Gy from a ^137^Cs source irradiator. At 15 weeks after transplantation of CD133^+^ cells, 3 × 10^4^ irradiated JEX28 cells were inoculated intraperitoneally into IBMI-humanized mice. Peripheral blood samples were collected every three weeks post-infection. Spleen and intestinal lymph node tissues were obtained after euthanizing the mice. Mice were anesthetized and euthanized upon reaching any of the following humane endpoints: (i) body weight decreased to less than 70% of their maximum weight, or (ii) 83 days had elapsed since HTLV-1 infection. All infections were performed in a Biosafety Level P2A laboratory in accordance with the guidelines of Kansai Medical University.

### 2.7. DNA Purification and Proviral Load Measurement

Genomic DNA was extracted from peripheral blood or tissues using a *Quick*-DNA Microprep Kit (Zymo Research, Irvine, CA, USA) following the manufacturer’s protocol. PVL was measured by quantitative PCR, as previously described [25]. Briefly, the primers and probes are designed to target HTLV-1 pX and human b-globin (HBB). The PVL was calculated as follows: [(copy number of pX)/(copy number of HBB/2)] × 100 (%).

### 2.8. Flow Cytometric Analyses

Peripheral blood or single-cell suspensions from spleen and lymph node tissues were incubated with fluorochrome-conjugated antibodies for 15 min at 4 °C. Tax-tetramer staining was performed according to the manufacturer’s instructions. Prior to flow cytometric analysis, propidium iodide was added to the cells at a final concentration of 1 µg/mL for viability assessment. Absolute cell numbers were quantified using AccuCount Ultra Rainbow Fluorescent Particles (Spherotech, Lake Forest, IL, USA) following the manufacturer’s protocol. Samples were analyzed using an FACS Canto II flow cytometer (BD Biosciences, San Jose, CA, USA), and data were processed with FlowJo software (version 10.9.0; BD Biosciences).

### 2.9. Statistical Analysis

Statistical analyses were performed using GraphPad Prism version 10.5.0. Longitudinal data were analyzed using a mixed-effects model (REML) with time and strain as fixed effects and individual mice as a random effect. Multiple comparisons were corrected using Sidak’s test. For time points at which data were available for only one group, those data were excluded from the statistical analysis. For comparisons between two groups, the Mann–Whitney U test was used. All individual mouse data points were plotted over time.

## 3. Results

### 3.1. Humanized NOG-HLA-A02 Transgenic Mice May Suppress HTLV-1 Proviral Load Increase After Infection

**Figure 1:**
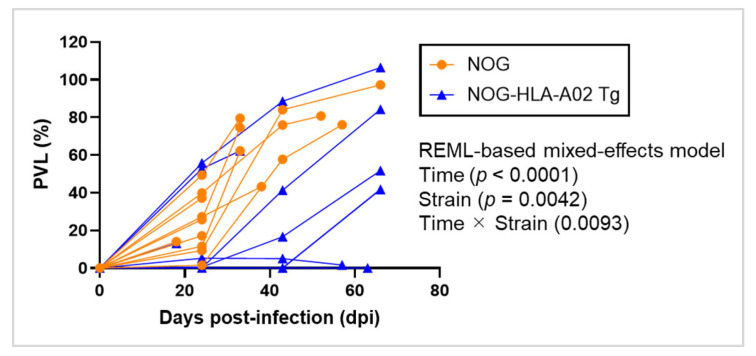
Humanized NOG-HLA-A02 transgenic mice suppress the increase in HTLV-1 proviral load after infection. Humanized NOG mice (*n* = 10) and humanized NOG-HLA-A02 transgenic mice (*n* = 9) were infected with HTLV-1. Peripheral blood was collected at regular intervals to measure PVL. *p*-values for time, strain, and their interaction were determined using an REML-based mixed-effects model and are shown in the figure. Differences between the two groups at individual time points were assessed with multiple comparisons corrected using Sidak’s test. Data were obtained from a single experiment. REML, restricted maximum likelihood.

To evaluate the impact of HLA-A02:01 expression on HTLV-1 infection, we compared the PVL over time between humanized NOG-HLA-A02 transgenic mice and humanized NOG mice after infection. An REML-based mixed-effects model revealed significant effects of time (*p* < 0.0001), strain (*p* = 0.0042), and their interaction (*p* = 0.0093) on PVL. The predicted mean PVL in the humanized NOG-HLA-A02 transgenic strain (27.64) was significantly lower than in the humanized NOG strain (63.53), with a difference of 35.89 (95% CI: 12.93–58.85) (Figure 1). Despite the significant time × strain interaction, no statistically significant differences in PVL were observed between the two strains at individual time points. This suggests that the overall interaction effect may reflect divergent temporal trends in PVL dynamics rather than consistent differences at specific time points. These results indicate that HLA-A02:01 expression may contribute to the suppression of PVL in vivo, particularly through its influence on the longitudinal course of infection.

### 3.2. Humanized NOG-HLA-A02 Transgenic Mice Suppress the Expansion of HTLV-1-Infected CD4^+^ T Cells After Infection

**Figure 2:**
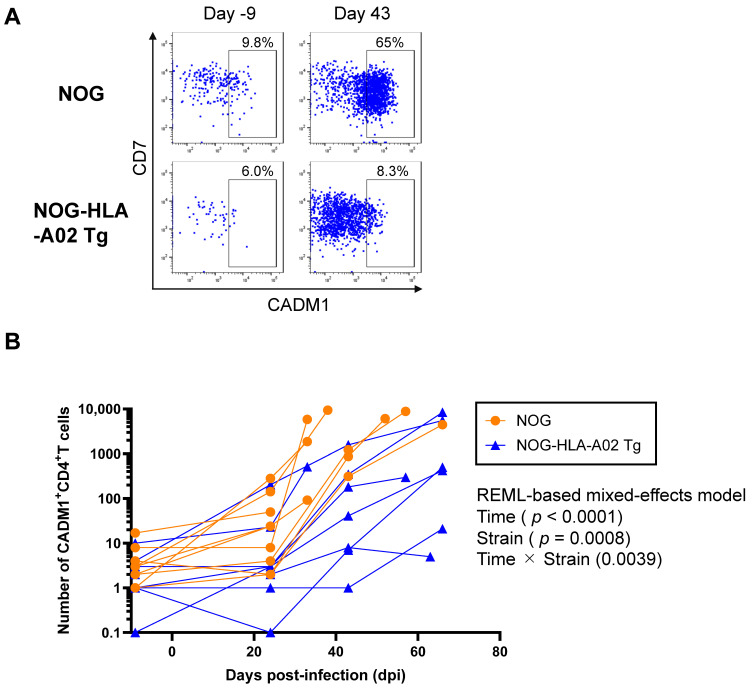
Humanized NOG-HLA-A02 transgenic mice suppress the expansion of HTLV-1-infected CD4^+^ T cells after infection. (**A**) Humanized NOG mice (*n* = 10) and humanized NOG-HLA-A02 transgenic mice (*n* = 9) were infected with HTLV-1. Peripheral blood was collected at multiple time points, and flow cytometric staining for CD4 and CADM1 was performed to identify HTLV-1-infected CD4^+^ T cells. Representative flow cytometry plots are shown. (**B**) The number of CADM1^+^ CD4^+^ T cells in peripheral blood was measured over time. The same mice used in panel A were analyzed in panel B. *p*-values for time, strain, and their interaction were determined using an REML-based mixed-effects model and are shown in the figure. Differences between the two groups at individual time points were assessed with multiple comparisons corrected using Sidak’s test. Data were obtained from a single experiment. REML, restricted maximum likelihood.

Next, we investigated whether humanized NOG-HLA-A02 Tg mice suppress the increase in HTLV-1-infected cells after infection. CADM1 is a well-established marker of HTLV-1-infected CD4^+^ T cells (Figure 2A) [26]. In this study, CADM1-high cells were gated as HTLV-1-infected cells according to a previously reported method [27]. Although CD7 expression is regarded as an indicator of ATL disease progression, it is not relevant to the gating strategy used here, and HTLV-1-infected cells were found in both CD7^+^ and CD7^−^ populations among CADM1^+^ cells. Therefore, we performed an REML-based mixed-effects model analysis to evaluate longitudinal changes in the number of CADM1^+^CD4^+^ T cells in peripheral blood. The mice used in Figure 2B are the same as those used in Figure 2A. Significant main effects were observed for both time (*p* < 0.0001) and strain (*p* = 0.0008), as well as a significant time × strain interaction (*p* = 0.0039), indicating that the temporal dynamics differed between the humanized NOG and NOG-HLA-A02 Tg strains (Figure 2B). The predicted mean number of CADM1^+^CD4^+^ T cells was significantly higher in the humanized NOG strain than in the humanized NOG-HLA-A02 Tg strain (difference: 2105; 95% CI: 1016–3194). However, post hoc comparisons at each time point did not reveal statistically significant differences between the two strains (all *p* > 0.05), suggesting that the strain difference became evident only when considering the overall temporal trend rather than individual time points.

### 3.3. Humanized NOG-HLA-A02 Transgenic Mice Tend to Exhibit Increased Frequencies of Tax-Specific CD8^+^ T Cells After Infection

**Figure 3:**
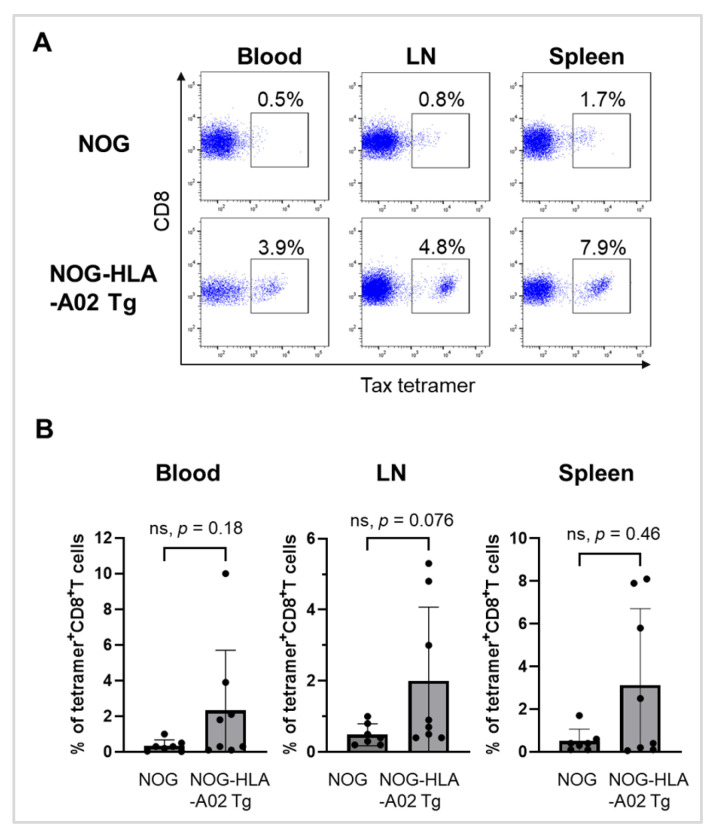
HTLV-1-infected humanized NOG-HLA-A02 transgenic mice tend to exhibit increased frequencies of Tax-specific CD8^+^ T cells. (**A**) Humanized NOG mice (*n* = 7) and humanized NOG-HLA-A02 transgenic mice (*n* = 8) were infected with HTLV-1. Mice were sacrificed and analyzed at a defined experimental endpoint: either (i) when body weight decreased to less than 70% of maximum weight or (ii) 83 days after infection. The endpoint at day 83 was chosen because only one humanized NOG mouse remained alive at approximately 12 weeks post-infection, considering the limited survival of the mice and the experimental schedule. Peripheral blood, lymph nodes, and spleen were collected, and cells were stained with HLA-A*02:01/Tax tetramers for flow cytometric analysis. Representative flow cytometry plots gated on CD8^+^ T cells are shown. (**B**) Frequencies of Tax-tetramer-positive cells among CD8^+^ T cells in each tissue are plotted. Analysis was performed at the same defined endpoint. Seven NOG mice and eight NOG-HLA-A02 transgenic mice were included in this panel. Statistical analysis (*p*-values) was performed using the Mann–Whitney U test. Data were obtained from a single experiment. LN, lymph nodes; ns, not significant.

To evaluate the induction of Tax-specific CD8^+^ T cells in HTLV-1-infected NOG-HLA-A02 Tg mice, we analyzed the frequency of HLA-A02:01/Tax-tetramer-positive cells among CD8^+^ T cells. Tax-specific CD8^+^ T cells were detected in the blood, lymph nodes, and spleen of both humanized NOG mice and humanized NOG-HLA-A02 Tg mice (Figure 3A). The frequencies of these cells were generally higher in humanized NOG-HLA-A02 Tg mice than in humanized NOG mice, with a notably higher trend observed in the lymph nodes (Figure 3B). However, the differences between the two mouse groups were not statistically significant at the defined experimental endpoint (either when body weight decreased to less than 70% of maximum weight or at 83 days post-infection, chosen due to limited survival of humanized NOG mice). These results suggest that humanized NOG-HLA-A02 Tg mice may mount a more robust Tax-specific CTL response.

### 3.4. Prolonged Survival of Humanized NOG-HLA-A02 Transgenic Mice Following HTLV-1 Infection

**Figure 4:**
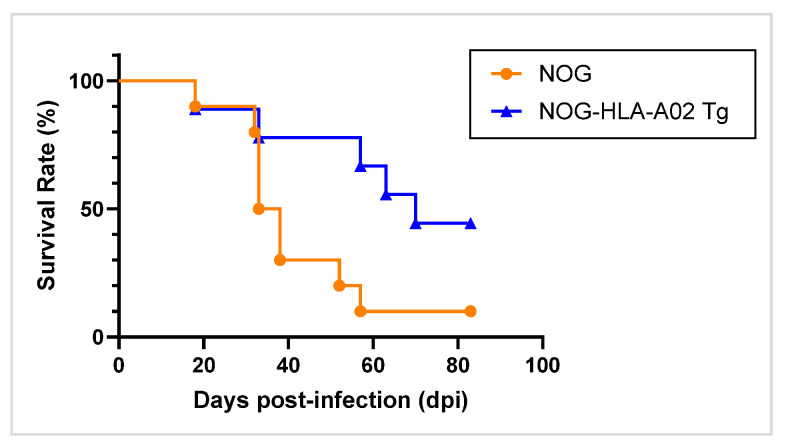
Prolonged survival of humanized NOG-HLA-A02 transgenic mice following HTLV-1 infection. Kaplan–Meier survival curves of HTLV-1-infected humanized NOG mice (*n* = 10) and humanized NOG-HLA-A02 transgenic mice (*n* = 9). Statistical analysis (*p*-values) was performed using the log-rank test. *p* < 0.05 is statistically significant. Data were obtained from a single experiment. HTLV-1, human T-cell leukemia virus type 1.

To determine whether the expression of HLA-A02:01 affects overall survival following HTLV-1 infection, we monitored the survival of humanized NOG-HLA-A02 Tg mice and humanized NOG mice over time after viral challenge. Kaplan–Meier survival analysis demonstrated that humanized NOG-HLA-A02 Tg mice had a significantly longer survival period compared to parental NOG mice (Figure 4). Statistical analysis using the log-rank test revealed a significant difference between the two groups (*p* = 0.033), indicating that the expression of HLA-A02:01 confers a survival advantage in HTLV-1-infected mice.

## 4. Discussion

In this study, we demonstrate for the first time that the transgenic expression of human HLA-A02:01 in an HTLV-1-infected humanized mouse model induces Tax-specific CD8^+^ T-cell responses, resulting in effective suppression of HTLV-1 infection in vivo. This provides direct experimental evidence that HLA-A02:01-restricted CD8^+^ T cells play a pivotal role in controlling HTLV-1 replication and pathogenesis.

Compared with non-human primate models, humanized mouse models offer several advantages, including lower cost, fewer facility restrictions, and ease of handling. However, one major limitation of conventional humanized mouse models is the incomplete reconstitution of the human immune system, which impairs their ability to faithfully recapitulate human immune responses. These models exhibit a persistent, abnormal expansion of infected cells (Figure 2B), contrasting with the infection pattern typically observed in humans, where infected cells expand during the early phase but subsequently reach a steady state [28]. These findings suggest an imbalance between viral replication and host antiviral immunity in the current humanized mouse systems.

The development of MHC-restricted T cells requires proper thymic education. Humanized BLT (bone marrow–liver–thymus) mice, generated through implantation of human fetal liver and thymus tissues under the renal capsule and intravenous or intra-bone marrow injection of CD34^+^ HSCs, have been shown to mount functional CTL responses against tumors and viral pathogens [29,30,31]. However, the generation of BLT mice is technically demanding and raises ethical concerns. As an alternative, humanized NOG-HLA-A02 Tg mice, which express HLA-A02:01 on thymic epithelial cells, have been shown to support Epstein–Barr virus (EBV)-specific cytolytic activity [20,32].

In our study, humanized NOG-HLA-A02 Tg mice exhibited a trend toward higher frequencies of Tax-tetramer^+^ CD8^+^ T cells compared to conventional NOG mice after infection (Figure 3B). This suggests that expression of human MHC class I in the thymic environment may promote the development of CD8^+^ T cells with more effective HLA-restricted TCRs. However, these differences were not statistically significant, and given the limited sample size and measurements at a single experimental time point, caution is warranted in interpreting this trend.

Although we did not investigate Tax expression in humanized NOG-HLA-A02 Tg mice, HTLV-1-infected cells in humanized NOG mice did not express Tax in vivo but did so under ex vivo culture conditions. This suggests that, similar to infected cells in humans [33], Tax expression in infected cells may be suppressed in humanized mice to evade CTL responses. In patients, Tax is thought to be intermittently expressed in only a small fraction of infected cells [34]. In our previous study, we observed an inverse correlation between the frequency of Tax-tetramer^+^ CD8^+^ T cells and PVL in PBMCs from HTLV-1-infected humanized NOG mice [25]. This result suggests that Tax-specific CD8^+^ T cells contribute to the suppression of HTLV-1 infection. Furthermore, in the present study, humanized NOG-HLA-A02 Tg mice exhibited reduced PVL and limited expansion of infected cells compared with humanized NOG mice after HTLV-1 infection (Figure 1 and Figure 2B). These findings further support the possibility that Tax-specific CD8^+^ T-cell responses contribute to suppression of HTLV-1 replication in vivo.

First, a limitation of this study is that we did not directly assess whether Tax-tetramer^+^ CD8^+^ T cells in our model can lyse HTLV-1-infected target cells. Nevertheless, previous studies have shown that Tax-specific CD8^+^ T cells from HTLV-1-infected individuals efficiently lyse autologous CD4^+^ T cells expressing Tax in a perforin-dependent manner [33], and CTL responses against dengue virus [19] and EBV [20,32] have been observed in humanized mouse models. Collectively, these findings suggest that humanized mice are capable of mounting CTL responses against Tax, indicating that further functional analyses in our model are warranted.

Second, the mechanism by which HLA-restricted Tax-tetramer^+^ CD8^+^ T cells develop in conventional humanized NOG mice lacking HLA expression remains unclear. One possible explanation is that dendritic cells derived from human hematopoietic stem cells migrate to the thymus and contribute to the development of HLA-restricted CD8^+^ T cells.

Third, although survival was prolonged in humanized NOG-HLA-A02 Tg mice following HTLV-1 infection compared to conventional humanized mice (Figure 4), it was not sufficient to completely prevent mortality associated with viral dissemination. This limitation may be attributed to the lack of functional CD4^+^ T-cell help and insufficient antibody production, as murine MHC-restricted CD4^+^ T cells cannot adequately support CTL responses or promote class switching in B cells. Previous studies have reported that humanized mice expressing both human MHC class I and II molecules develop more robust CTL responses [35,36]. Therefore, introducing human MHC class II genes into NOG-HLA-A02 Tg mice may further enhance immune responses against HTLV-1 by facilitating effective CD4^+^ T-cell help.

In conclusion, we established a humanized mouse model expressing human HLA-A*02:01 that enables the evaluation of cellular immune responses against HTLV-1. Our findings highlight the critical role of Tax-specific CD8^+^ T-cell responses in suppressing HTLV-1 propagation. This model offers a valuable tool for investigating HTLV-1 immunopathogenesis and evaluating the efficacy of therapeutic strategies such as vaccines and immune checkpoint blockade therapies.

## Data Availability

The original contributions presented in this study are included in the article. Further inquiries can be directed to the corresponding author.

## Abbreviations

The following abbreviations are used in this manuscript:

HTLV-1	human T-cell leukemia virus type 1
ATL	adult T-cell leukemia/lymphoma
HAM/TSP	HTLV-1-associated myelopathy/tropical spastic paraparesis
CTLs	cytotoxic T lymphocytes
Tg	transgenic
PVL	proviral load
ACs	asymptomatic carriers
HSCs	hematopoietic stem cells
TCRs	T-cell receptors
MHC	major histocompatibility complex
MNCs	mononuclear cells
IBMI	intra-bone marrow injection
HBB	human b-globin
REML	restricted maximum likelihood
EBV	Epstein–Barr virus
